# Assessment of the heavy metal bioremediation efficiency of the novel marine lactic acid bacterium, *Lactobacillus plantarum* MF042018

**DOI:** 10.1038/s41598-019-57210-3

**Published:** 2020-01-15

**Authors:** Fatma A. Ameen, Amira M. Hamdan, Moustafa Y. El-Naggar

**Affiliations:** 1 Botany and Microbiology Department, Faculty of Science, Alexandria University, Alexandria, Egypt; 2 Oceanography Department, Faculty of Science, Alexandria University, Alexandria, Egypt

**Keywords:** Applied microbiology, Water microbiology

## Abstract

Heavy metal pollution is one of the most serious environmental and human health risk problem associated with industrial progress. The present study was conducted with the goal of isolation and characterization of metal-resistant lactic acid bacteria (LAB) from the Alexandrian Mediterranean Seacoast, Egypt, with their possible exploitation in metal remediation. *Lactobacillus plantarum* MF042018 exhibited high degree of resistance, up to 500 and 100 ppm, to both nickel and chromium, respectively, with multiple antibiotic resistance (MAR) index above 0.5. In an attempt to improve chromium removal by *L. plantarum* MF042018, Plackett-Burman followed by Box-Behnken statistical designs were applied. An initial Cr^2+^ concentration of 100 ppm and inoculum size of 3% presented the best conditions for the accumulation of chromium by *L*. *plantarum* MF042018. The study was also navigated to assess the biosorption capacity of *L*. *plantarum* MF042018, the maximum uptake capacity (*q*) of both Cd^2+^ and Pb^2+^ was recorded at pH 2.0 and a temperature of 22 °C after 1 hr. The biosorption process of Cd^2+^ and Pb^2+^ was well explained by the Langmuir isotherm model better than the Freundlich isotherm. Furthermore, the results revealed that the use of *L*. *plantarum* MF042018 is an effective tool for the treatment of hazardous metal-polluted battery-manufacturing effluent. Therefore, the present study implies that *L*. *plantarum* MF042018 can be applied as a promising biosorbent for the removal of heavy metals from industrial wasterwaters.

## Introduction

As a consequence of disastrous anthropogenic activities, the discharge of hazardous heavy metal pose devastating threat to environmental safety and subsequently lead to severe concerns on human health worldwide^1^. Nowadays, several approaches have been successfully developed to use magnetic nanoparticles (MNPs), such as magnetic chitosan/graphene oxide (MCGO) composite, and ultrasonic irradiation for the synthesis of metal organic frameworks (MOFs) for the removal of heavy metals from contaminated environments^2,3^.

In this context, the expansion of new technologies has led to the evolution of “Bioremediation” as a powerful alternative tool to reduce the adverse consequences of the tremendous accumulation of heavy metals^4^. Bioremediation; using bacteria, fungi, yeast and algae, is an efficient, cost-effective and environmentally friendly strategy that has recently received great attention to tackle heavy metal contamination^5^. Recently, the use of microorganisms especially fungi as potential biosorbents for the removal of heavy metals from industrial wastewater effluents has been extensively investigated^6–8^.

Lactic acid bacteria (LAB) are Gram-positive bacteria, that have gained Generally Recognized As Safe (GRAS) status by the US Food and Drug Administration (FDA) and have a strong record of safe application as probiotic supplements^9^. Recently, several strains of LAB have generated much attention to their potential use in the removal of heavy metals for protection of human health^10–13^.

A considerable number of studies have reported the application of *Lactobacillus plantarum* strains for decontamination of heavy metals in aqueous solutions and foodstuffs, such as fruit and vegetable juices, as well as improving the nutritional value of food^14–16^. However, to our knowledge, there is limited information on the use of LAB for the bioremediation of metal-contaminated industrial effluents. Consequently, the main objective of the current study was to explore the effectiveness of novel metal-resistant marine LAB isolated from the Alexandrian Mediterranean Seacoast, Egypt, as a potential agent for the treatment of hazardous heavy metals from industrial wastewater. In addition, the study extended to explore the optimal conditions for maximum metal removal by the novel marine *Lactobacillus plantarum* using Plackett-Burman and Box-Behnken statistical designs. Furthermore, attempts were also taken to evaluate the efficiency of the application of *Lactobacillus plantarum* in metal bioremediation of battery-manufacturing waste.

## Results and Discussion

### Metal resistance profile of marine LAB

A total of 12 marine LAB were initially selected for screening of metal-resistance efficiencies against; Cr^2+^, Ni^2+^, Cd^2+^ and Pb^2+^ (from 10 to 600 ppm). All LAB candidates showed noticeable elevated minimum inhibitory concentrations (MIC) value ≤ 600 ppm against Ni^2+^, whereas only LAB 9 isolate exhibited remarkably high potential of Cr^2+^ resistance with MIC value ≤ 150 ppm (Data not shown). However, on the other hand, Cd^2+^ and Pb^2+^ were highly toxic and lethal to all tested LAB strains where growth was notably suppressed.

### Identification of metal-resistant LAB 9 isolate

On the basis of morphological, biochemical characteristics and 16S rRNA gene sequence analysis, LAB 9 isolate was identified as *Lactobacillus plantarum* MF042018. Similarity percentage showed 99% homology to *Lactobacillus plantarum* HIF81 (Accession Number KU748635.1) available in the NCBI database (http://www.ncbi.nlm.nih.gov). Subsequently, the sequence of 16S rRNA gene of LAB 9 was submitted to the GenBank under the Accession Number of LC381759.1.

### Antibiotic resistance pattern of *Lactobacillus plantarum* MF042018

*L. plantarum* MF042018 exhibited acquired resistance against 11 tested antibiotics; Ampicillin/Sulbactam, Cefotaxime, Norfloxacin, Piperacillin/tazobactam, Cefoxitin, Ciprofloxacin, Tobramycin, Cefuroxime, Piperacillin, Ceftazidime and Cefaclor, with MAR index value of 0.55 (Data not shown). Several studies revealed that the genes encoding both heavy metal and antibiotic resistance are often located together on the same plasmid^17^. Furthermore, resistance to antibiotics indicates that the isolated LAB strains would be able to withstand the undesirable high concentrations of antibiotics occasionally present in the environment^11^. Moreover, Mishra *et al*.^18^ reported that such metal and antibiotic resistances are adopted by the spread of R-factors rather than by mutation and/or natural selection under metal stress environmental conditions.

### Bioaccumulation of Ni^2+^ and Cr^2+^ by *L*. *plantarum* MF042018

**Figure 1:**
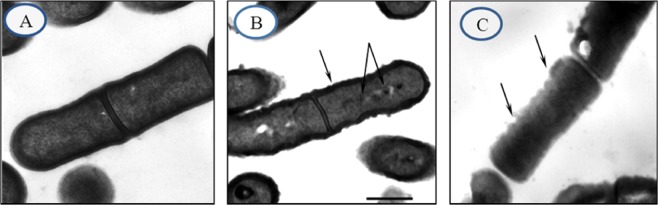
Transmission electron micrographs of *L. plantarum* MF042018 before and after metal exposure **(A)** Control untreated cells; **(B)** Ni-treated cells; **(C)** Cr-treated cells. (Scale bar represents 200 nm). ❖Arrows show precipitates of metal particles on both cell surface and interior of the cells.

The percentage removal of Ni^2+^ and Cr^2+^ by *L. plantarum* MF042018 was calculated after 24 hrs of incubation and the obtained results revealed that *L. plantarum* MF042018 was able to efficiently remove Ni^2+^ and Cr^2+^ from the broth medium by 33.8 ± 0.8% and 30.2 ± 0.5%, respectively (Data not shown). Moreover, TEM micrographs of *L. plantarum* MF042018 revealed obvious changes in the cell surface morphology of both Ni- and Cr-treated cells, compared to the control cells, with the appearance of dense metal deposits uniformly adsorbed to the outer cell surface and accumulated inside the cells (Fig. 1).

**Figure 2:**
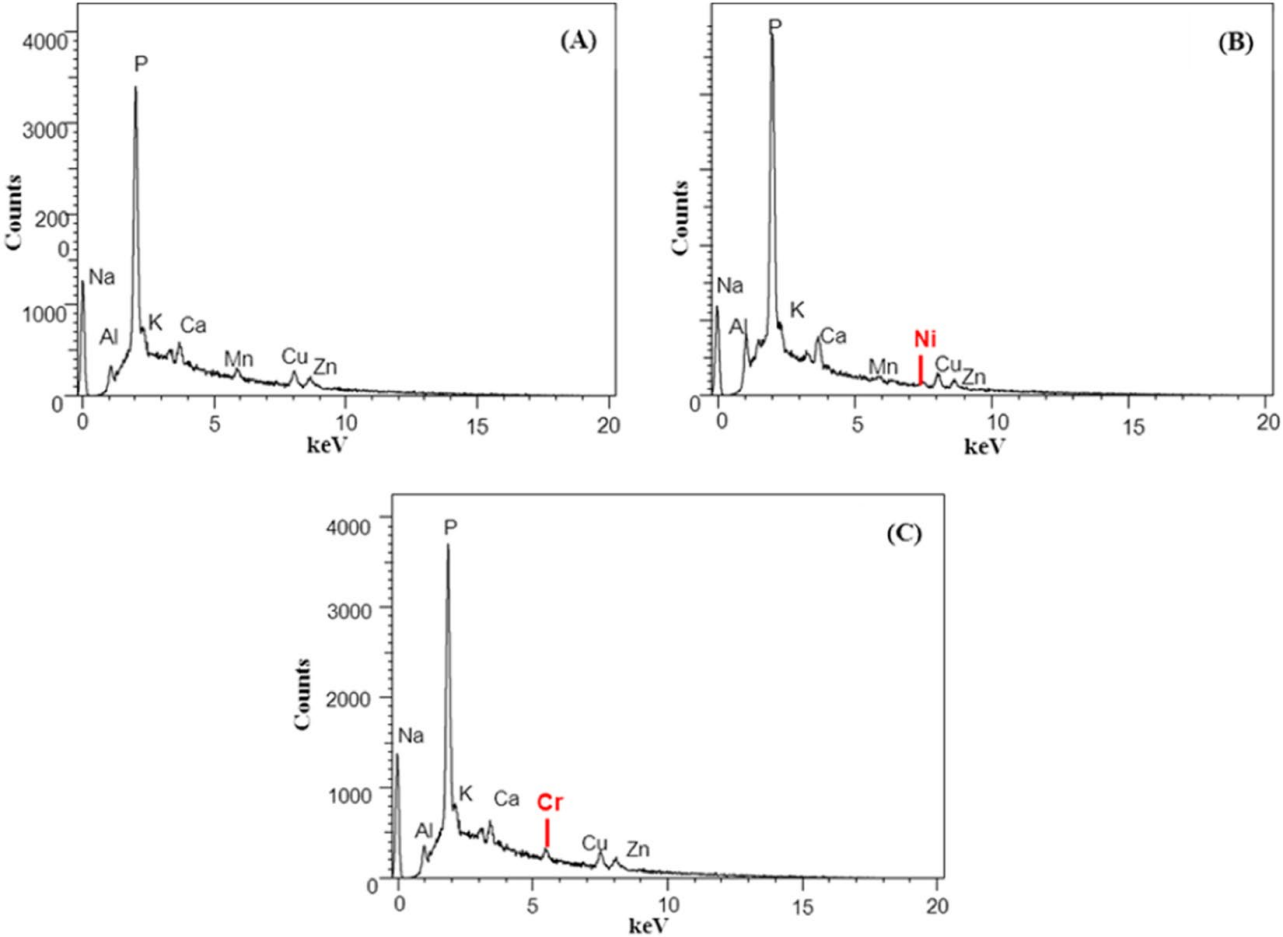
Energy dispersive X-ray (EDX) spectra of untreated cells (**A**), Ni-treated cells **(B**) and Cr-treated cells (**C**).

According to Wang^19^, there are two main mechanisms involved in bacterial resistance to Cr; biotransformation of Cr(VI) to Cr(III), that is less toxic, and biosorption, where bacteria trap the pollutant in its biomass. In addition, Monachese *et al*.^1^ reported the capability of a number of species belonging to the genus *Lactobacillus* to bind metals, including Cr, and to detoxify them from different heavy metals contaminated geographical areas. Moreover, concerning EDX analysis, no Ni or Cr signals could be detected in the control samples (Fig. 2A), whereas clear Ni and Cr peaks were observed at 7.5 and 5.6 keV, respectively, in Ni- and Cr-treated cells (Fig. 2B,C).

Recently, magnetically modified graphene oxide/chitosan/ferrite (GCF) and chitosan grafted graphene oxide (CS-GO) nanocomposite materials were successfully investigated as suitable adsorbents for efficient removal of Cr(VI) from industrial wastewaters^20–22^. Moreover, Samuel *et al*.^23^ developed graphene oxide/fungal hyphae (GO-FH) bio-nanocomposite material with maximum adsorption capacity of Cr(VI) (up to 212 mg/g) and excellent regeneration performance.

### Statistical optimization of Cr^2+^ removal by *L*. *plantarum* MF042018 using RSM

**Figure 3:**
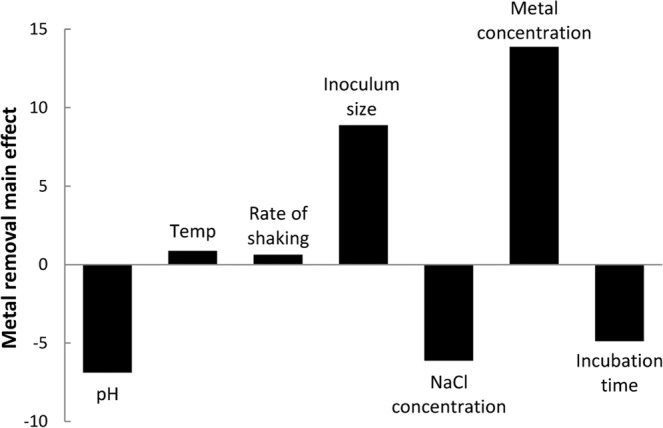
(Main) Effect of different independent variables on percentage metal removal by *L. plantarum* MF042018.

**Figure 4:**
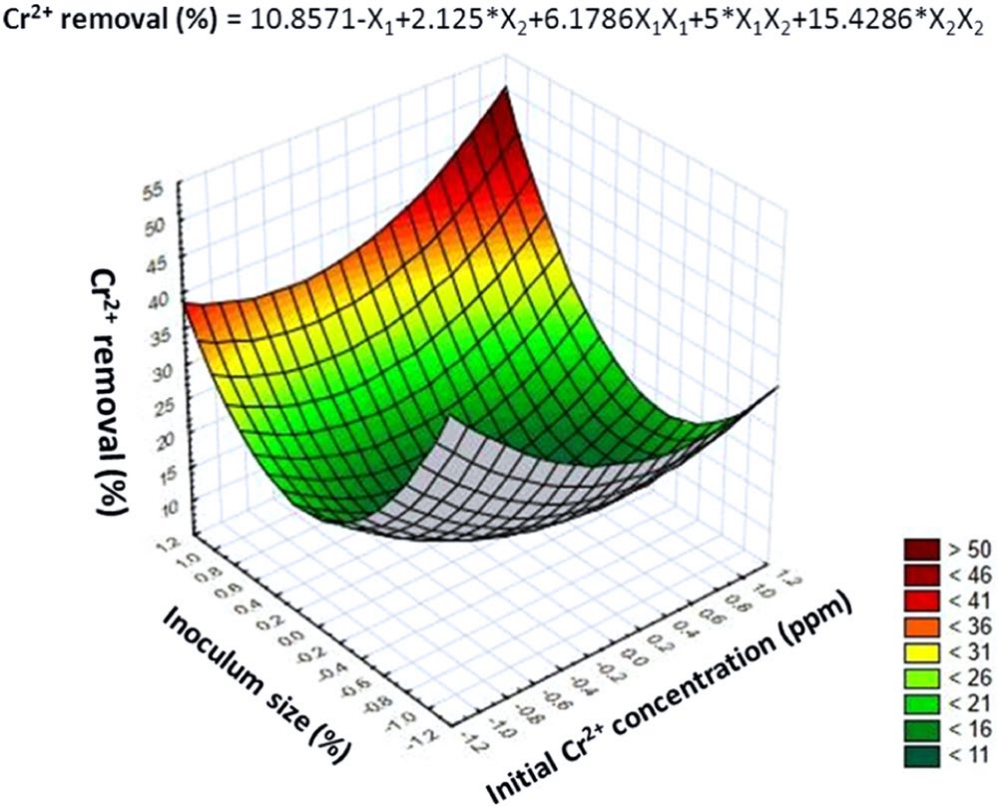
3-D response surface plot showing the effect of the interaction between initial Cr^2+^concentration (ppm) and inoculum size (%) on Cr^2+^ removal (%) by *L. plantarum* MF042018.

Plackett-Burman design was applied to allow reliable short listing of significant culture conditions affecting maximum metal removal. The results showed that, out of the 7 tested variables, the presence of high levels of both initial Cr^2+^ concentration and inoculum size in the culture medium had significant positive effects on metal removal, with confidence levels above 95% (*P* < 0.05), whereas the other tested variables gave insignificant negative effects toward metal removal (Table 1, Fig. 3). Furthermore, the regression coefficient (*R*^2^) value of the model was 0.998 that reflects an excellent fit of the model and is considered reliable as having a very high correlation^24^. Based on these results, the effect of initial Cr^2+^ concentration and inoculum size on percent removal of chromium was performed by Box-Behnken design, with all other variables kept at the lowest levels, and a 3-D surface graph was constructed (Fig. 4). The figure shows that the maximum percentage removal of chromium (~50%) can be achieved with the increase in both initial metal concentration (100 ppm) and inoculum size of 3%. This result is in accordance with the results reported by dos Reis *et al*.^25^, where maximum accumulation of Fe^3+^ by *Bacillus subtilis* UFLA SCF590 was attributed to the effect of lower pH value of 3.5 with high concentration of Fe^3+^ (0.75 ppm). Furthermore, the results obtained by El-Naggar *et al*.^26^ showed that the biosorption of Pb^2+^ by the marine red alga, *Gelidium amansii*, reached its maximum with the increase in initial Pb^2+^ concentration up to 200 mg/l, beyond which biosorption gradually decreased. On the contrary, Choińska-Pulita *et al*.^27^ reported that the efficiency of Cd^2+^ and Zn^2+^ biosorption by *Pseudomonas azotoformans* JAW1 is inversely proportional to initial metal concentration, which could be explained by the insufficient metal binding sites on microbial biomass. Thus, increasing biomass concentration causes an increase in contact surface area, leading to higher heavy metal removal efficiency^28^.

**Table 1:** ANOVA statistical analysis of metal removal using Plackett-Burman experiment.

Variables	Main Effect	Coefficients	Standard error	*t*-value	*P*-value	Confidence level (%)
Intercept	—	20.1875	—	—	—	
pH	−6.875	−3.4375	0.3125	−11	0.080	99.92
Temperature	0.875	0.4375	0.3125	1.4	0.394	99.60
Rate of Shaking	0.625	0.3125	0.3125	1.2	0.605	99.39
Inoculum Size	8.875	4.4375	0.3125	14.2	0.044	99.95
NaCl Concentration	−6.125	−3.0625	0.3125	−9.8	0.064	99.93
Incubation Time	13.875	−2.4375	0.3125	−7.8	0.081	99.91
Metal Concentration	−4.875	6.9375	0.3125	22.2	0.028	99.97

### Biosorption of cadmium and lead by *L. plantarum* MF042018

Kirillova *et al*.^29^ reported that studies on Pb and Cd are often conducted together, as both elements seem to react with bacterial species in similar ways. The potential binding capacity of *L. plantarum* MF042018 to remove Cd^2+^ and Pb^2+^ from aqueous solutions was evaluated under different reaction conditions. A contact time of 1 hr was recommended by Halttunen *et al*.^14^, wherein rapid binding phenomenon, due to high affinity of free metal ion binding sites, was observed across all studied *Lactobacillus* strains after 1 hr of incubation whereas prolonged exposure led to reduction in metal removal.

**Figure 5:**
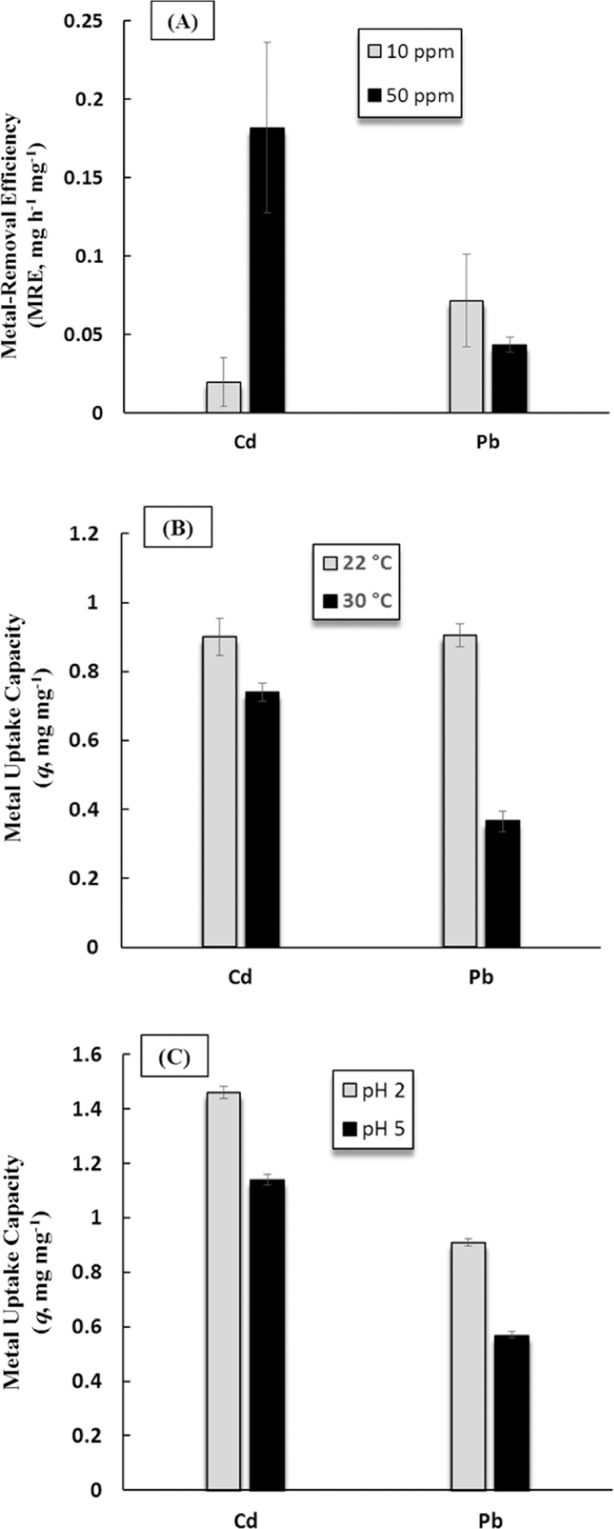
Effect of initial metal concentration (**A**), incubation temperature (**B**) and initial pH (**C**) on biosorption of Cd^2+^ and Pb^2+^ by *L. plantarum* MF042018.

The results revealed that both Cd^2+^ and Pb^2+^ removal efficiencies by *L. plantarum* MF042018 were concentration-dependent where maximum removal efficiencies (MRE) of Cd^2+^ (0.18 ± 0.054 mg Cd h^−1^mg^−1^) and Pb^2+^ (0.07 ± 0.03 mg Pb h^−1^mg^−1^) were recorded with initial metal concentrations of 50 ppm and 10 ppm, respectively (Fig. 5A). Whereas nearly negligible fractions of Cd and Pb were removed upon increasing metal concentration to 80 ppm, as similarly reported by Oves *et al*.^30^, where high metal concentrations lead to substantial decline in metal biosorption capacity due to the saturation of adsorption sites on the peptidoglycan layer and the lack of sufficient free binding sites. However, Samuel *et al*.^2^ showed high adsorptive potential (92%) of Pb^2+^, from an initial concentration of 50 ppm, using magnetic chitosan/graphene oxide (MCGO) composite material within 7 hrs. Moreover, Vilela *et al*.^31^ demonstrated the efficiency of graphene oxide-based microbots (GOx-microbots) for the removal of Pb^2+^ from contaminated water by adsorption process and the subsequent reusability of GOx-microbots and recovery of lead. On the other hand, graphene oxide/fungal hyphae (GO-FH) bio-nanocomposite material exhibited excellent performance of adsorption and regeneration of chromium(VI) up to 212.76 mg/g^23^.

Furthermore, monitoring environmental physicochemical parameters; pH and temperature, can significantly influence microbial binding of heavy metals^32^. The results showed that the highest uptake capacities (*q*) of Cd^2+^ (1.46 ± 0.022 mg Cd mg^−1^) and Pb^2+^ (0.91 ± 0.020 mg Pb mg^−1^) by *L. plantarum* MF042018 were achieved at 22 °C and pH 2 (Fig. 5B,C). Whereas, the removal of both Cd^2+^ and Pb^2+^ was almost negligible with increasing pH and/or temperature beyond pH 5 and/or 30 °C. The obtained results were in agreement with Sofu *et al*.^33^, where 20 °C was the optimum temperature for maximum removal of Fe(II) and Zn(II) by *Lactobacillus delbrueckii ssp bulgaricus* (Lb-12) and *Streptococcus thermophillus* (STM-7). In contrast, Halttunen *et al*.^34^ reported significant increase in Cd removal by *Lactobacillus rhamnosus* GG when the incubation temperature was raised to 37 °C. Furthermore, Yi *et al*.^35^ and Halttunen *et al*.^14^ indicated that, for *in vivo* practical use, LAB perform high Pb removal capacity at low pH that could be due to a higher number of phosphate groups available on the bacterial surface. Similarly, Hansen *et al*.^36^ revealed that acidic pH was optimum for maximum removal of As(V) by *Lessonia nigrescens*. However, Topcu and Bulat^37^ reported that the lowest level of removal for both cadmium and lead by *Enterococcus faecium* is typically observed at pH below 3 wherein a linear increase in metal removal took place with an increase in pH.

**Figure 6:**
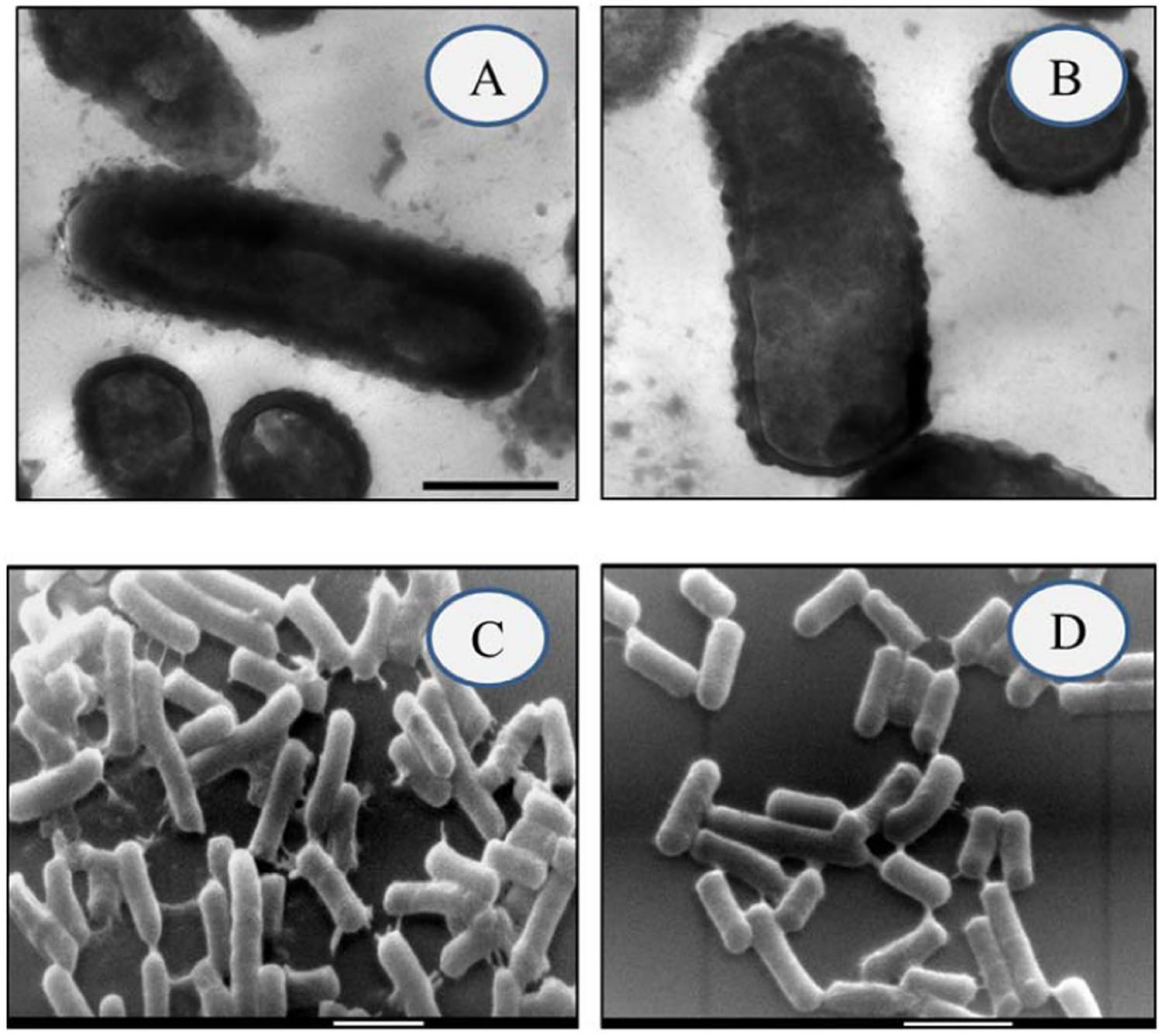
Electron micrographs of Cd and Pb biosorption by *L. plantarum* MF042018 biomass. (**A,B**) TEM micrographs after Cd and Pb binding, respectively. (Scale bar represents 200 nm). **(C,D)** SEM micrographs after Cd and Pb binding, respectively. (Scale bar represents 1 μm).

**Figure 7:**
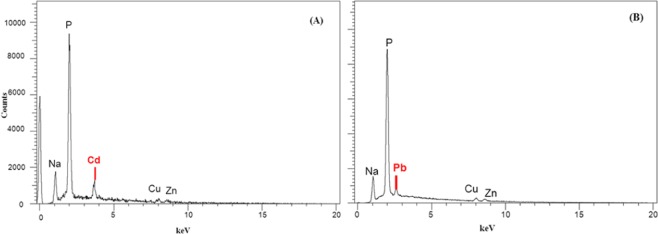
Energy dispersive X-ray (EDX) spectra of *L. plantarum* MF042018 biomass after Cd binding (**A**) and Pb binding (**B**).

Biosorption of Cd^2+^ and Pb^2+^ by *L. plantarum* MF042018 was verified by TEM and SEM examinations and EDX analysis as presented in Figs. 6 and 7. TEM micrographs clearly confirmed that both Cd^2+^ and Pb^2+^ formed visible deposits covering the cell surface reflecting high binding capacity (Fig. 6A,B). In addition, SEM micrographs revealed that exposure to Cd^2+^ and Pb^2+^ led to enormous aggregation of *L. plantarum* MF042018 cells (Fig. 6C,D) compared to the untreated cells, with no morphological changes. Furthermore, EDX analysis also detected additional Cd and Pb peaks in both Cd- and Pb-treated cells (Fig. 7A,B) due to biosorption, whereas no peaks were observed in the control sample (Fig. 2A).

Morphological alterations of *L. plantarum* MF042018 after Cd^2+^ and Pb^2+^ exposure, observed by TEM and SEM examinations, were in concordance with studies conducted by Halttunen *et al*.^38^ who found large deposits of lead on the surface of lyophilized *Bifidobacterium longum* 46 and *Lactobacillus fermentum* ME3 after lead binding. Zhai *et al*.^15^ mentioned that such phenomenon may be a form of self-protection by *Lactobacillus plantarum* CCFM8610 cells caused by the change in surface charge and the degeneration of surface proteins enhanced by Cd exposure and led to the aggregation of cells.

### Cd and Pb biosorption isotherms and kinetics

The Langmuir and Freundlich adsorption isotherm parameters for Cd^2+^ and Pb^2+^ biosorption by *L. plantarum* MF042018 biomass were calculated and presented in Table 2. It appears that the biosorption process of both metal ions was more consistent with the Langmuir isotherm model than the Freundlich isotherm model. The regression coefficient (*R*^2^) values of the Langmuir equation for both Cd^2+^ (*R*^2^ = 0.991) and Pb^2+^ (*R*^2^ = 0.957) conform better than those of the Freundlich equation. In addition, *R*_L_ values for Cd^2+^ (0.526) and Pb^2+^ (0.613) are greater than “0” indicating that the sorption equilibrium is favorable with the Langmuir model as reported by Khan *et al*.^39^. These findings are in accordance with the results of Chakravarty and Banerjee^40^ and Zhai *et al*.^15^ that suggest the contribution of both electrostatic reaction and complex formation together with the high affinity of cadmium binding to the bacterial biomass.

**Table 2:** The Langmuir and Freundlich isotherm parameters for Cd^2+^ and Pb^2+^ biosorption onto *L. plantarum* MF042018 biomass.

	Langmuir model				Freundlich model		
	*q*_*max*_	*K*_*L*_	*R*^2^	*R*_L_	*K*_*f*_	*n*	*R*^2^
Cd	18.18	0.018	0.991	0.526	1.77	1.40	0.982
Pb	11.21	0.063	0.957	0.613	0.53	2.56	0.912

Where; qmax, maximum adsorption capacity (mg/g); KL and Kf, Langmuir and Freundlich adsorption constants (l/mg), respectively; R2, regression coefficient; n, the adsorption intensity; RL, Separation factor.

### Removal of heavy metals from battery-manufacturing effluent by *L. plantarum* MF042018

The potency of *L. plantarum* MF042018 to facilitate the removal of toxic heavy metals from industrial effluents was examined. After 1 hr exposure of the effluent waste to *L. plantarum* MF042018 enclosed in a dialysis tube, the percentage removal of metal ions was calculated. The used cells achieved a 100% removal of Ni^2+^, Cr^2+^, Cd^2+^ and Pb^2+^ ions, indicating the successful ability of *L. plantarum* MF042018 to remove a mixture of heavy metals from the industrial effluent within 1 hr only. Furthermore, applying the microbial mass hanged in a dialysis tube is considered a significant criterion, to facilitate the collection and separation of cells from the treated industrial effluent for further use^41^.

## Materials and Methods

### Sample collection and isolation of marine LAB

A total of 20 sediment and seawater samples were aseptically collected from different stations along the Alexandrian Mediterranean Seacoast, Egypt, and kept at 4**°**C for further studies. Collected samples were used for isolation of LAB by the spread plate technique using de Man, Rogosa and Sharpe (MRS) agar media (Difco, USA) incubated anaerobically at 30 °C for 48 hrs^42^. Selected Gram positive isolates with catalase negative activity were preserved on MRS agar slants at 4**°**C for routine use or maintained in MRS broth supplemented with 30% glycerol at −80**°**C for long-term storage.

### Screening for potential metal-resistant LAB isolates

Metal solutions, Ni(NO_3_)_2_, Pb(NO_3_)_2_, Cd(NO_3_)_2_ and Cr(NO_3_)_2_ (Sigma-Aldrich, USA), were sterilized by filtration through 0.45μm Millipore bacterial filters (Advantec, Tokyo, Japan). Metal-resistant profiles of LAB isolates were conducted on MRS agar plates amended with metals concentrations (up to 600 ppm) and incubated under anaerobic conditions at 30 °C for 1–3 days. The minimum inhibitory concentrations (MICs) were assessed by determining the lowest metal concentrations that completely inhibited LAB growth^39^. The higher-growth-exhibiting isolates were selected as potential metal-resistant LAB isolates for further studies.

### Biochemical and molecular characterization of metal-resistant LAB isolates

Potential metal-resistant LAB isolates were biochemically characterized by API 50 CHL test kits (BioMérieux, Marcy I´Etoile, France) according to the manufacturer’s instructions and interpretation of results was performed using the computer-aided database API-WEB™V.5.0 software as mentioned by Khalil *et al*.^43^. Furthermore, selected isolates were identified by 16S rRNA gene sequencing, following Hamdan *et al*.^44^, analyzed in the Genbank DNA database using the online tool (BLAST) at NCBI (http://www.ncbi.nlm.nih.gov/BLAST/), and deposited in the DNA Data Base of Japan (DDBJ) for serial accession numbers.

### Bioremediation of heavy metals by LAB isolates

Metal-resistant LAB isolates were cultured in MRS broth and incubated at 30 °C for 24 hrs on a rotary shaker at 120 rpm. Triplicate sets of MRS broth with initial concentration of 500 ppm for Ni or 100 ppm for Cr were inoculated with 1% freshly prepared LAB isolates and incubated at 30 °C. After 24 hrs, cells were harvested by centrifugation at 6,000 rpm for 10 min to remove metal-bound bacterial cells and supernatants were analyzed by atomic absorption spectrophotometer (Shimadzu 6800, Japan) to determine residual metal ions concentrations. The average values were used to measure the percentage of metal removal according to the following equation^15^:

$${\rm{Metal}}\,{\rm{removal}}\,( \% )=\frac{{{\rm{C}}}_{{\rm{i}}}-{{\rm{C}}}_{{\rm{f}}}}{{{\rm{C}}}_{{\rm{i}}}}\times {\rm{100}}$$

Where; “C_i_” and “C_f_” are the initial and final concentrations of metal, respectively.

LAB isolates with the highest percentage of metal removal were selected for further experiments.

### Optimization of Cr^2+^ removal by Response Surface Methodology (RSM)

#### Plackett-Burman design (PBD)

Plackett–Burman experiment^45^ was performed to select the most significant variable(s) that influence the process of Cr^2+^ removal under metal stressed culture conditions. Seven independent variables were examined (evaluated) in two levels, low (−1) and high (+1) levels, including: initial pH (X_1_), incubation temperature (X_2_), rate of shaking (X_3_), inoculum size (X_4_), NaCl concentration (X_5_), incubation time (X_6_) and initial metal concentration (X_7_) (Table 3). The selected variables were organized in 8 different trials, performed in triplicate sets, and the effect of each variable on the percentage of metal removal was determined based on the following equation^27^:

$$Y={\beta }_{0}+\varSigma {\beta }_{1}{X}_{1}$$

Where; “Y” is the response (Cr^2+^ removal, %), “β_0_” is the model intercept and “β_1_” is the linear coefficient, and “X_1_” is the level of the independent variable.

**Table 3:** Independent variables for the evaluation of Cr^2+^ removal by selected LAB isolate using Plackett–Burman design.

Independent Variables	Codes	Experimental levels	
		−1	+1
Initial pH	X_1_	5	9
Incubation Temperature (°C)	X_2_	20	40
Rate of Shaking (rpm)	X_3_	90	150
Inoculum Size (%)	X_4_	0.5	1.5
NaCl Concentration (%)	X_5_	0	10
Incubation Time (hr)	X_6_	20	40
Initial Metal Concentration (ppm)	X_7_	25	75

Where; −1 = low level; + 1 = high level.

#### Box–Behnken design

Based on the results of PBD, two significant variables; Cr^2+^ concentration (ppm) and inoculum size (%), with high influence on Cr^2+^ removal were selected for further optimization using Box-Behnken design^46^. In triplicate sets, each variable was tested in 15 experimental trials at three different levels; low (−1), middle (0) and high (+1), as shown in Table 4. The interaction effect between the percentages of Cr^2+^ removal and the significant independent variables were estimated using the following second order polynomial equation^17^:

$${\rm{Y}}={{\rm{\beta }}}_{0}+{{\rm{\beta }}}_{1}{{\rm{X}}}_{1}+{{\rm{\beta }}}_{2}{{\rm{X}}}_{2}+{{\rm{\beta }}}_{12}{{\rm{X}}}_{1}{{\rm{X}}}_{2}+{{\rm{\beta }}}_{11}{{\rm{X}}}_{1}^{2}+{{\rm{\beta }}}_{22}\,{{\rm{X}}}_{2}^{2}$$

Where; “Y” is the predicted response (Cr^2+^ removal, %), “β_0_” is the regression coefficient, “β_1_” and “β_2_” are the linear coefficients, “β_12_” is the interaction coefficient, “β_11_” and “β_22_” are the quadratic coefficients, and “X_1_” and “X_2_” are the independent variables.

**Table 4:** The levels of variables selected for the Box-Behnken optimization design.

Variables	Codes	−1	0	+1
Metal Concentration (ppm)	X_1_	50	75	100
Inoculum Size (%)	X_2_	1	2	3

#### Metal biosorption assay

LAB was anaerobically incubated at 30 °C for 48 hrs in MRS broth and cells were harvested by centrifugation at 6,000 rpm for 10 min and washed three times with sterile ultra-pure Milli-Q (MQ) water. Biosorption experiments were performed following Halttunen *et al*.^14^ with slight modifications. Briefly, cell pellets [30 mg ml^−1^ (wet weight)] were re-suspended in 5 ml sterile MQ water supplemented with various concentrations (10, 50 and 80 ppm) of Cd^2+^ or Pb^2+^ and incubated at 30 °C for 1 hr. Furthermore, temperature and pH effects on metal binding capacity of LAB isolates were assessed by adjusting the pH of the metal-amended solutions to pH 2.0, 5.0 and 7.0, using 1 N NaOH or HCl, and incubation temperatures were carried out at 22, 30 and 37 °C. Samples were collected, centrifuged and concentrations of residual, non-adsorbed, metals were measured in supernatants using atomic absorption spectrophotometer. LAB pellets suspended in metal-free MQ water were considered as negative controls, whereas positive controls of only Cd or Pb solutions were included in each experiment. All experiments were performed in triplicates and average values were used to calculate the metal uptake capacity (*q*, mg mg^−1^)^47^ and the metal-removal efficiency (MRE, mg h^−1^ mg^−1^)^48^ by LAB isolates as follows:

$$q=\frac{{C}_{i}-{C}_{f}}{W}\times {\rm{V}}$$

Where; “V” is the reaction volume (l), and “W” is the total cell biomass (mg) used in the reaction mixture.

$${\rm{MRE}}=\frac{{C}_{i}-{C}_{f}}{({t}_{f}-{t}_{i})M}$$

Where; “t_i_” and “t_f_” are the initial and final contact time, respectively, and “M” is the cell biomass (mg).

#### Antibiotic sensitivity and resistance pattern of LAB isolates

Antibiotic susceptibility assay of metal-resistant LAB isolates was performed on MRS agar plates seeded with 1% overnight cultured LAB against 20 different commercial antibiotics; Neomycin (30 μg), Erythromycin (15 μg), Trimethoprim/Sulfamethoxazole (25 μg), Ampicillin/Sulbactam (20 μg), Cefaclor (30 μg), Ciprofloxacin (5 μg), Amikacin (30 μg), Gentamicin (10 μg), Imipenem (10 μg), Rifampicin (30 μg), Ceftazidime (30 μg), Azithromycin (15 μg), Cefotaxime (30 μg), Norfloxacin (10 μg), Piperacillin/tazobactam (110 μg), Cefoxitin (30 μg), Piperacillin (100 μg), Cefuroxime (30 μg), Levofloxacin (5 μg) and Tobramycin (10 μg) (Oxoid, UK) using standard disc diffusion method^49^. Antibiotic resistant profile of LAB isolates was assessed according to the measured diameter (mm) of inhibition zone around the disc and the ™Multiple Antibiotic Resistance (MAR) index for each isolates was calculated following Jain *et al*.^50^:

$${\rm{MAR}}\,{\rm{index}}=\frac{a}{b}$$

Where; “a” is the number of resistant antibiotics, and “b” is the total number of antibiotics used.

#### Biosorption isotherm and kinetics of LAB isolates

The metal biosorption capacity at equilibrium (*q*_e_) was calculated using the following equation^15^;

$${q}_{e}=\frac{{V(C}_{i}-{C}_{e})}{M}$$

Where; “C_e_” is the metal concentration at equilibrium.

Both the Langmuir and the Freundlich isotherm models were applied to the metal uptake data of the LAB isolates as described by Khan *et al*.^39^ and Yi *et al*.^35^. The Langmuir isotherm model is represented using the following equation;

$${q}_{e}=\frac{{q}_{{\rm{\max }}}{K}_{L}{C}_{e}}{1+{K}_{L}{C}_{e}}$$

Where; “*q*_max_” is the maximum adsorption capacity (mg/g), and “*K*_L_” is the Langmuir adsorption constant (l/mg).

In order to express the feasibility of the Langmuir isotherm model, a dimensionless separation factor (*R*_L_), or equilibrium parameter, was calculated using the following equation;

$${R}_{L}=\frac{1}{1+{K}_{L}{C}_{i}}$$

The Freundlich isotherm model is represented using the following equation;

$${q}_{e}={K}_{F}{{C}_{e}}^{1/n}$$

Where; “*K*_*F*_” is the Freundlich adsorption constant (l/mg), and “*n*” is the adsorption intensity.

#### Electron Microscopy and Energy Dispersive X-ray (EDX) analysis

In order to verify the adverse effects of metal stress on cell structure and morphology and investigate the possible localization of accumulated metals within LAB isolates, collected cell pellets of both metal-free (control) and metal-treated isolates were fixed with 2.5% (w/v) glutaraldehyde buffered in 0.1 mol l^−1^ phosphate buffer (pH 7.2). For Scanning Electron Microscopy (SEM; JEOL JSM 5400 LV, japan) and Transmission Electron Microscopy (TEM; JEOL JSM 5300, Japan), fixed samples were prepared as described by Khan *et al*.^39^ and Zhai *et al*.^15^, respectively. Furthermore, detection of metal elements present in LAB cells was carried out using Energy Dispersive X-ray (EDX) (JEOL JSM 6360 LA, Japan).

#### Biosorption of heavy metals from battery-manufacturing effluent

Based on testing the capability of the selected LAB to remove toxic heavy metal ions from the prepared metal solutions, biosorption experiment was performed using an industrial waste effluent to confirm the capability to remove a mixture of heavy metals from such waste. Effluent samples were collected from the outlet pipes of a battery-manufacturing factory in Alexandria city, Egypt, and stored at 4 °C. The effluent was filter sterilized using 0.45μm Millipore bacterial filters and was initially adjusted to pH 5.0 using 1 N HCl. Cells of LAB were harvested by centrifugation, washed with sterile MQ water, confined in dialysis tubing and suspended into effluent samples at an inoculum level of 2% (w/v). Triplicate tubes were incubated at 22 °C for 1 hr and effluent tubes devoid of bacterial inoculum were used as controls. Residual metal ions concentrations were measured by atomic absorption spectrophotometer.

#### Statistical analysis of data

All experiments were carried out in triplicates and the results were expressed as the mean ± standard deviation (SD). The obtained data were subjected to one-way analysis of variance (ANOVA) followed by Student’s *t*-test to estimate *t*-value, *P-*value and confidence levels and results were considered statistically significant when *P* < 0.05. All statistics were performed using Statistical Package for the Social Sciences (SPSS) program (Version 12.0, SPSS Inc., Chicago, IL). The three-dimensional (3-D) surface plots were generated using STATISTICA software (Version 5.0, StatSoft Inc., Tulsa, USA).

## Conclusion

The preliminary obtained results revealed that *Lactobacillus plantarum* MF042018 has high tolerance against Ni^2+^ and Cr^2+^ with potential bioremediation capacity and antibiotic-resistant criteria. Moreover, efficient binding ability of Cd^2+^ and Pb^2+^, from aqueous solutions was achieved at pH 2 and low incubation temperature (22 °C), and closest fit the Langmuir isotherm model. Based on these findings, further investigations should focus on the feasibility of *Lactobacillus plantarum* MF042018 biomass as an appropriate, inexpensive tool for detoxification of heavy metal-contaminated environments and/or foodstuffs that would be a reliable area for industrial-scale applications.
